# Polylactic Acid/Polyaniline Nanofibers Subjected to Pre- and Post-Electrospinning Plasma Treatments for Refined Scaffold-Based Nerve Tissue Engineering Applications

**DOI:** 10.3390/polym15010072

**Published:** 2022-12-24

**Authors:** Yongjian Guo, Rouba Ghobeira, Sheida Aliakbarshirazi, Rino Morent, Nathalie De Geyter

**Affiliations:** Department of Applied Physics, Research Unit Plasma Technology (RUPT), Faculty of Engineering & Architecture, Ghent University, Sint-Pietersnieuwstraat 41, B4, 9000 Ghent, Belgium

**Keywords:** PLA/PAni, nanofibers, APPJ plasma treatment, DBD plasma treatment, PC-12 cells, neurite extension

## Abstract

Composite biopolymer/conducting polymer scaffolds, such as polylactic acid (PLA)/ polyaniline (PAni) nanofibers, have emerged as popular alternative scaffolds in the electrical-sensitive nerve tissue engineering (TE). Although mimicking the extracellular matrix geometry, such scaffolds are highly hydrophobic and usually present an inhomogeneous morphology with massive beads that impede nerve cell-material interactions. Therefore, the present study launches an exclusive combinatorial strategy merging successive pre- and post-electrospinning plasma treatments to cope with these issues. Firstly, an atmospheric pressure plasma jet (APPJ) treatment was applied on PLA and PLA/PAni solutions prior to electrospinning, enhancing their viscosity and conductivity. These liquid property changes largely eliminated the beaded structures on the nanofibers, leading to uniform and nicely elongated fibers having average diameters between 170 and 230 nm. After electrospinning, the conceived scaffolds were subjected to a N_2_ dielectric barrier discharge (DBD) treatment, which significantly increased their surface wettability as illustrated by large decreases in water contact angles for values above 125° to values below 25°. X-ray photoelectron spectroscopy (XPS) analyses revealed that 3.3% of nitrogen was implanted on the nanofibers surface in the form of C–N and N–C=O functionalities upon DBD treatment. Finally, after seeding pheochromocytoma (PC-12) cells on the scaffolds, a greatly enhanced cell adhesion and a more dispersive cell distribution were detected on the DBD-treated samples. Interestingly, when the APPJ treatment was additionally performed, the extension of a high number of long neurites was spotted leading to the formation of a neuronal network between PC-12 cell clusters. In addition, the presence of conducting PAni in the scaffolds further promoted the behavior of PC-12 cells as illustrated by more than a 40% increase in the neurite density without any external electrical stimulation. As such, this work presents a new strategy combining different plasma-assisted biofabrication techniques of conducting nanofibers to create promising scaffolds for electrical-sensitive TE applications.

## 1. Introduction

The 20th century has ended with a massive surge of attention to electrospinning after discovering its ability to generate fibers from various polymers. This technique is actually associated with several appealing strengths, such as its affordability, versatility, simplicity and capacity to adjust the diameter of the produced fibers from tens of nanometers up to hundreds of micrometers [1]. The extremely high surface area, enhanced porosity and unique fibrous morphology of electrospun structures have made them good candidates in a variety of applications, such as sensors, catalysts supports and optical devices [2]. In particular, electrospun polymeric nanofibers have transfigured the rapidly evolving tissue engineering (TE) field due to their great mimicry of the geometrical features of the extracellular matrix where cells reside [3]. Considerable improvements in cell adhesion, proliferation, migration and differentiation were actually detected on 3D nanofibers compared to 2D flat surfaces [4].

When taking a look at the extensive electrospinning literature, one can notice that biodegradable poly(α-hydroxy ester) based polymeric nanofibers are the most widely used in TE applications. This specific polymer group is commonly selected as base material given its renowned biocompatibility, tailorable mechanical properties and biodegradability rate. A notable supremacy is spotted for polylactic acid (PLA) based scaffolds in several TE fields, such as nerve, blood vessel, bone, liver, tendon and cartilage regenerative applications [5,6]. In fact, PLA has been approved by the Food and Drug Administration (FDA) for its safe medical use in humans given its good biocompatibility and degradation into non-toxic components [6]. Moreover, PLA is soluble in most organic solvents and readily processable into a very good nanofiber quality [7]. Starting from these advantages, a new class of composite nanofibers based on biopolymers, such as PLA, polyglycolic acid (PGA) and polycaprolactone (PCL), but combined with conducting polymers are nowadays specifically attracting the interest of the scientific community as important materials in the rapidly emerging technology advancements. A variety of conductive polymers are being used for this purpose such as polyaniline (PAni) [8], polypyrrole (PPy) [9], poly(3,4-ethylenedioxythiophene) polystyrene sulfonate (PEDOT:PSS) [10], carbon nanotubes (CNTs) [11] and reduced graphene oxide (rGO) [12]. The resulting conductive composite scaffolds have gained great popularity for their use in electrical-sensitive TE applications to enhance, amongst others, nerve regeneration [13]. In fact, these scaffolds do not only inherit the biocompatibility and biodegradability of the bulk biopolymer but also trigger improved performances of nerve and other types of electrical-sensitive cells in conjunction with [11,12,14] or without [8,15,16] external electrical stimulation. In fact, the main function of central and peripheral nerves is to transmit electrical signals. Therefore, electrospun nanofibers exhibiting conductive properties were shown to have the ability to enhance the proliferation, differentiation and migration of nerve and glial cells and connect the interrupted electrical transmission between the ends of damaged nerves and spinal cord thereby promoting neural regeneration [17]. For instance, Shu et al. have revealed that electrospun PLA/PPy nanofibers exhibited a significantly higher electrical conductivity than PLA nanofibers which could enhance the conduction of the electrical signal in a rat spinal cord injury model, thus triggering an improved functional recovery [18]. Wu et al. have demonstrated that the addition of conductive CNTs to poly(p-dioxanone) nanofibers could greatly enhance the differentiation ability of human adipose-derived mesenchymal stem cells into Schwann cell-like cells when electrically excited [19]. Another study has additionally reported that electrospun PCL nanofibers coated with PPy could positively affect the proliferation of Schwann cells [20]. Next to the differentiation and enhanced behaviors of glial cells, Prabhakaran et al. have also revealed a greatly improved viability and proliferation of nerve stem cells when cultured on electrospun PAni-PLA scaffolds compared to pure PLA nanofibers. Moreover, the cells could extend longer neurites when seeded on the conductive PLA/PAni nanofibers [14]. Among the used inherently conducting polymers (ICPs), PAni actually offers some extra advantages, including its unique doping mechanism, low biotoxicity, high environmental stability, facile synthesis from low-cost monomers and good electrical conductivity. PAni has different oxidation states, including the fully reduced leucoemeraldine base (LEB), the half-oxidized emeraldine base (EB) and the fully oxidized pernigraniline base (PB) [21]. The EB state of PAni or PAni EB is blue in color but can turn into a green-colored conducting emeraldine salt (ES) when doped by a protonic acid, such as the organic acid camphorsulfonic acid (CSA) [22]. The biocompatibility of PAni has undergone some debates because of its non-biodegradability, which could incite, on the long term, some undesired side effects such as chronic inflammation as a result of debris formation [23]. Nonetheless, Kamalesh et al. have successfully proven the long term in vivo biocompatibility of PAni films post-subcutaneous implantation in rats for 90 weeks. No abnormalities in the neighboring tissues nor signs of toxicity or unwanted inflammatory responses were detected [24]. Mattioli-Belmonte et al. also conducted in vivo studies on different polymeric implants including PAni and have not found any adverse impact on surrounding tissues, such as inflammation or tumor formation [25]. Next to these in vivo studies, the in vitro biocompatibility of pure PAni or PAni blended with other polymers has been recognized with several cell lines, such as PC-12 cells, H9c2 cardiac myoblasts, L929 murine fibroblasts and rat nerve stem cells. Although showing a good level of biocompatibility, reservations still exist for future clinical translations since loosely arranged fibrous tissue was visualized 24 weeks after subcutaneous implantation of PAni films in rats [23]. Nevertheless, this problem can be counteracted by blending PAni with other biodegradable and biocompatible polymers like PLA [17]. Moreover, extra measures could be taken when reaching the clinical stage, such as purifying PAni via repetitive de-protonation and re-protonation cycles. In fact, Humpolicek et al. have shown that this approach decreases the cytotoxicity of PAni [26]. PLA/PAni composites present a typical combination of a biopolymer/conducting polymer as they have been employed in different TE fields, including bone [27], nerve [14] and heart [28,29]. Nonetheless, two main issues are associated with this type of scaffolds. On the one hand, despite their good biocompatibility, biodegradability and electro-activity, most PLA-based scaffolds are highly hydrophobic because of their low surface energy. This resulting inert surface property induces low cell affinity, such as poor adhesion and non-uniform spreading [30]. On the other hand, when compared to conventional non-conductive polymers, PAni is hard to be electrospun mainly because of its rigid backbone and low solubility, which leads to a low-quality beaded nanofiber morphology [21]. 

To overcome the first issue related to the low surface energy, a surface modification grafting polar functionalities on the final composite product can be performed. In fact, polar groups are known to bind bioactive molecules such as proteins on which the cell receptors attach thus initiating proper cell-material interactions [31]. Among the different surface engineering techniques, non-thermal plasma treatment is attracting a considerable interest, as in addition to its eco-friendly character, its process parameters can be delicately fine-tuned to prevent damage of delicate nanofibrous structures. When a non-thermal plasma is sustained in air, N_2_, Ar, O_2_ or NH_3_, oxygen and/or nitrogen-containing functional groups are introduced onto the substrates [32,33]. This was recurrently shown to efficiently change the surface wettability of scaffolds from hydrophobic to hydrophilic states and prominently improves the affinity of different cell types, such as Schwann cells and PC-12 cells [34,35]. Interestingly, the action of plasma treatment is restricted to a few nanometers in depth, which preserves the bulk properties inherited from the used biopolymers [36].

The second issue, associated with low-quality beaded PLA/PAni nanofibers, can cause a problem in nerve TE in particular. Unlike other TE fields, nerve TE is actually distinctive and hierarchical. A complete reconstruction of the neuronal network involves more than the initial adhesion, migration and proliferation of neurons, which are analogous phases to other cell types. The subsequent synaptic communication between neurons is the key leading to the establishment of functional neural circuits that determine the ability of signal transmission from and to different parts of the body [37]. This latter process can be influenced by many physical cues of the neuron-resided extracellular microenvironment. In particular, the guidance from surface geometrical features is of critical importance to nerve cell performances, including neurite formation and contact [38,39]. Within this context, obtaining flawless electrospun scaffolds with appropriate morphology for nerve TE is sometimes a very challenging process, especially when using difficult to spin polymers such as PAni. Beads, one of the common defects occurring in electrospun scaffolds, can lead to an irregular and disjointed nanofiber morphology [40]. When neurons are seeded on beaded nanofibrous scaffolds, the potential impediment of neurite outgrowth, although not yet reported, cannot be excluded. Typically adopted strategies to eliminate beaded structures in electrospinning are the addition of salts to the polymer solutions or the enhancement of the solvent polarity by using systems containing highly toxic solvents like hexafluoro-2-isopropanol (HFIP—especially when PAni is present) [29,41,42,43,44]. However, these approaches often involve extra costs, safety/toxicity concerns as well as non-eco friendliness. The use of electrospinning solutions with high polymer concentrations can also potentially lead to bead-less nanofibers. Nonetheless, an accompanying undesired increase of the fiber diameter will occur [7]. In fact, neurons were shown to sense and respond to surface nanotopography, with an astonishing sensitivity to changes of a few nanometers [45]. As such, an alternative environmentally friendly method is strongly needed to improve the electrospinnability of polymer solutions. Within this context, researchers have recently found that the use of an atmospheric pressure plasma jet (APPJ) is an effective method leading to the fabrication of bead-less nanofibers. It should be noted that, in contrast to the above-mentioned plasma treatment that is directly applied on the surface of the electrospun scaffolds, the APPJ treatment is a prepositive procedure applied on the polymeric solutions prior to their electrospinning. Such an APPJ treatment mainly results in the degradation of the solvent molecules instead of the polymeric macromolecules, thus contributing to a higher solution conductivity and polarity, which is responsible for the improved electrospinnability [46,47]. The earliest related exploration was in 2010 when a poly(ethylene oxide) (PEO) solution was plasma-treated pre-electrospinning leading to nanofibrous scaffolds exhibiting fewer beads and a more homogeneous diameter distribution than the fibers electrospun from untreated solutions [48]. Afterwards, solutions of biodegradable polymers, including PLA [49,50] and PCL [51], have also been modified with an APPJ, and similar noticeable improvements illustrated by uniform and bead-less morphologies were detected. 

Acknowledging the above, this work presents a combinatorial strategy encompassing successive pre- and post-electrospinning plasma treatments of PLA, PLA/PAni EB and PLA/PAni ES solutions and their resultant electrospun nanofibers, respectively. To the best of our knowledge, no study has so far adopted this dual plasma-assisted biofabrication and functionalization of PAni-containing nanofibers that can offer great benefits enhancing nerve regeneration. Prior to electrospinning, the solutions were subjected to an APPJ treatment sustained in argon, as this particular treatment was already successfully applied to enhance the electrospinnability of other types of polymer solutions [50,51]. The untreated and plasma-treated solutions were characterized by assessing their pH, viscosity and conductivity. After electrospinning, the nanofibrous scaffolds ensuing from the different polymeric solutions were exposed to a dielectric barrier discharge (DBD) plasma sustained in nitrogen at medium-pressure [33]. The physicochemical changes induced by the different plasma treatments were carefully evaluated and characterized using scanning electron microscopy (SEM), static water contact angle (WCA) goniometry and X-ray photoelectron spectroscopy (XPS). Finally, the bio-responsive properties of the untreated and different plasma-treated nanofibrous scaffolds were investigated making use of PC-12 cells. This cell line was purposely chosen giving its well-established capacity of neural differentiation and neurite extension in appropriate environments, thus representing an ideal in vitro model for a proof-of-concept in nerve TE studies. An extensive comparative study of the cell-surface affinity and neurite outgrowth between different combinations of untreated, APPJ-treated (surface morphology influence), DBD-treated (surface chemistry and wettability influence) and PAni-containing (conductivity influence) scaffolds was performed. 

## 2. Materials and Methods

### 2.1. Preparation of Polymer Solutions

PLA granules (molecular weight (M_w_) _=_ 230,000 g/mol) were purchased from Goodfellow (Hamburg, Germany). PAni EB (M_w_ = 56,000 g/mol), (1S)-(+)-10-camphorsulfonic acid (CSA), chloroform (CHCl_3_, purity = 99.5%), and *N, N*-dimethylformamide (DMF, purity = 99.8%) were all purchased from Sigma-Aldrich. In this work, PLA, PLA/PAni and protonic acid-doped PLA/PAni:CSA solutions were prepared. To do so, PLA pellets and/or PAni powders were dissolved in a mixture of CHCl_3_ and DMF (8:2 *v*/*v*). The concentrations of PLA and PAni were 6 *w*/*v*% and 0.2 *w*/*v*%, respectively. The CSA-doped PAni solution was prepared by mixing PAni and CSA powder in a mole ratio of 2:1 (two CSA molecules for every four aromatic rings of PAni).

### 2.2. APPJ Treatment of Polymer Solutions

**Figure 1:**
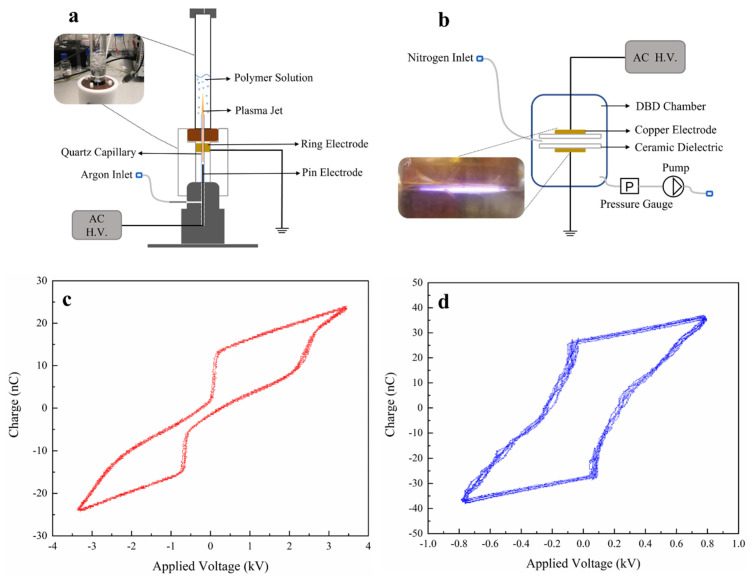
Schematical representation of the (**a**) APPJ set-up used to treat polymer solutions and (**b**) DBD set-up used to treat nanofibrous scaffolds (the insets in (**a**) and (**b**) show photographs of the APPJ treatments of polymer solution and the DBD treatment of the resultant nanofibrous scaffold, respectively); Q-V Lissajous figures of the (**c**) APPJ and (**d**) DBD discharge.

Prior to electrospinning, the prepared solutions were APPJ treated by means of an in-house made device that can submerge the plasma jet in the polymer solutions under treatment as schematically represented in Figure 1a. In brief, the device is composed of a tungsten rod acting as a high voltage electrode and a copper ring serving as a grounded electrode. The rod, having a diameter of 1 mm and a half-sphere-shaped tip, was tightly placed in a cylindrical quartz capillary with inner and outer diameters of 1.5 and 3.0 mm, respectively. The ring electrode having an inner diameter 0.2 mm larger than the outer capillary diameter was fixed in a way surrounding the capillary at a distance of 18 mm above the tip of the rod electrode. Before plasma treatment, argon (Alphagaz 1, Air Liquide) was sent through a capillary tube to the reactor chamber being a tubular glass sample holder that is perforated on its bottom to slide in and encircle the end of the quartz capillary. Thereafter, 10 mL of the prepared polymer solution was poured into the chamber. By switching a 50 kHz AC customer-made power source on, Ar plasma was generated in the inter-electrode gap and outflew, as a result of the Ar flow in the capillary, into the reactor chamber so that the plasma afterglow treats the polymer solution. For all performed treatments, the amplitude of the applied voltage, argon flow rate and plasma exposure time were fixed at 3.4 kV, 0.5 slm (standard liters per minute) and 3 min, respectively. To calculate the discharge power of the APPJ, the discharge voltage V(t) applied to the reactor was measured using a 1000:1 high voltage probe (Tektronix P6015A), which was connected to the pin electrode. Additionally, the charge Q(t) stored on the electrodes was determined by measuring the voltage V_c_(t) over a capacitor of 10 nF placed in series with the plasma jet. The measured V(t) and Q(t) were then recorded with a PC oscilloscope (PicoScope 3204A). The charge Q(t) was represented as a function of the discharge voltage V(t) in order to obtain the Q-V Lissajous figure, which is shown in Figure 1c. In the figure, the electrical energy consumed per voltage cycle is equal to the area enclosed by the Lissajous figure [52]. The discharge power P was then calculated by multiplying the electrical energy by the frequency of the power source (50 kHz) and was found to be equal to 3.2 W.

### 2.3. Liquid Properties Characterization of Polymeric Solutions

Some liquid properties, such as pH, conductivity and viscosity, were measured pre- and post-APPJ treatment to spot the plasma-induced modifications of the polymeric solutions. The pH of the solutions was assessed using a FiveEasy pH meter (Mettler Toledo) equipped with a probe purposely designed for non-aqueous solutions (InLab Science Pro-ISM pH probe). The solution conductivity was determined by means of a FiveEasy conductivity meter (Mettler Toledo) with an InLab 720 conductivity probe having a range of 0.1–500 μS/cm. The viscosity of the solutions was identified using a DV2T EXTRA 10 viscometer (Brookfield). The reported results represent the average of 3 values obtained from 3 independent measurements of different samples.

### 2.4. Fabrication of Nanofibrous Scaffolds

The prepared polymeric solutions were used to fabricate nanofibrous mats by means of the electrospinning technique. To do so, the bottom-up Nanospinner 24 machine (Inovenso), consisting of a high voltage metallic nozzle vertically placed below a grounded stainless steel cylindrical collector, was employed. Firstly, 5 mL of the solution to be electrospun was collected in a plastic syringe that was placed in a syringe pump (NE-300 Just Infusion) used to regulate the solution flow rate to 1 mL·h^−1^. When a polymer drop reached the metallic nozzle, a high voltage of 25 kV was applied leading to the deposition of fibers on cover slips (diameter: 12 mm) taped on an aluminium sheet rolled onto the cylindrical collector. The nozzle-to-collector distance was fixed at 20 cm throughout the process. The electrospinning process was conducted at room temperature with a relative humidity varying between 40% and 50%. For the plasma-modified solutions, the electrospinning process was conducted within 30 min after the APPJ treatment.

### 2.5. DBD Treatment of the Nanofibrous Scaffolds

Nitrogen plasma treatment was carried out on the electrospun mats in a parallel-plate DBD reactor schematically represented in Figure 1b. A detailed description and electrical characterization of the setup can be found in previous work [53]. Briefly speaking, the DBD reactor comprises two circular parallel copper electrodes (diameter: 38 mm) covered by ceramic (Al_2_O_3_) plates (thickness: 0.7 mm) acting as dielectric barriers. The distance between the two ceramic plates is kept constant at 4 mm. The upper electrode is connected to a 50 kHz AC power source, while the lower electrode is connected to ground. Before the treatment, the electrospun nanofibrous scaffolds were placed on the center of the lower ceramic plate. The treatment procedure began with the reactor being pumped down to a pressure of about 0.3 kPa using a rotary vane pump. The reactor was then refilled with nitrogen at a flow rate of 3.0 slm until a pressure between 80 kPa and 90 kPa was achieved. The pressure was kept in this range for 3 min in order to carry out an exhaustive purge of the residual air and improve the purity of nitrogen in the reactor. Subsequently, the reactor chamber was pumped down to 5.0 kPa, and the nitrogen flow was reduced to 1.0 slm. Using these latter conditions, the plasma treatment was performed for various exposure times. Similar to the characterization method of the APPJ discharge described in Section 2.2, the discharge power of the DBD was calculated using the Q-V Lissajous figure shown in Figure 1d and was found to be equal to 1.9 W.

### 2.6. Characterization of the Nanofibrous Scaffolds

The morphology of the nanofibrous scaffolds was visualized by imaging the samples using a JEOL JSM-6010 PLUS/LV scanning electron microscope. The SEM images were acquired with an accelerating voltage of 7 kV and a working distance of 10 mm after coating the samples with a thin layer of gold by means of a JEOL JFC-1300 Auto Fine coater. The average diameter of the nanofibers was calculated after measuring the size of 50 randomly chosen individual fibers on 3 different samples using ImageJ software (National Institutes of Health). 

To investigate the surface wettability of the nanofibrous mats, static WCAs were determined using a Laplace-Young curve fitting of the profile of a 2 µL distilled water drop deposited on the sample surfaces. The measurements were carried out using a Krüss Easy Drop optical system operating at room temperature. For each condition, the reported WCA result was obtained by averaging 6 values of 6 independent measurements taken on at least 2 samples.

The surface chemical composition of the different nanofibrous scaffolds was analyzed via XPS measurements. To do so, a PHI 5000 Versaprobe II spectrometer (ULVAC-Physical Electronics) equipped with a monochromatic Al Kα X-ray source (hν = 1486.6 eV) and operating at a power of 25 W (beam size of 100 μm) was employed. The pressure of the main XPS chamber was constantly kept below 10^−6^ Pa during the measurements. The emitted photoelectrons were detected with a hemispherical analyzer placed at an angle of 45° relative to the plane of the samples. Survey scans and high-resolution C1*s* and N1*s* spectra were recorded at pass energies of 187.85 eV (0.8 eV step size) and 23.5 eV (0.1 eV step size), respectively. The acquired survey scans (0–1100 eV) were then analyzed via Multipak software (version 9.6) to determine and quantify the present surface elements after applying a Shirley background subtraction with the relative sensitivity factors provided by the manufacturer of the instrument. The hydrocarbon component of the C1*s* spectrum (285.0 eV) was used as calibration of the energy scale. Curve fitting of the high-resolution C1*s* and N1*s* spectra was performed using the same software in order to identify the carbon-bonded groups. Gaussian−Lorentzian peak shapes were used for the deconvolution of the envelopes, and the full width at half maximum (FWHM) of each line shape was maintained below 1.6 eV. The survey scans and high-resolution spectra of all conditions were recorded on two different samples with four measurement points per sample.

### 2.7. PC-12 Cell Culture Tests on the Nanofibrous Scaffolds

To investigate neuron-like cellular interactions with the untreated and different plasma-treated nanofibrous mats, in vitro assays using rat PC-12 cells kindly provided by Prof. Dr. Leybaert (Ghent University, Belgium) were conducted. The cells were first cultured in a culture flask (T75) coated with 1 mL of collagen IV (Sigma-Aldrich) using Roswell Park Memorial Institute (RPMI) 1640 Glutamax containing 10% heat-inactivated horse serum, 5% heat-inactivated fetal bovine serum and 1% penicillin/streptomycin (Sigma-Aldrich). The components of the medium that was refreshed every 2 days were purchased from Thermo Fisher Scientific unless otherwise mentioned. Before cell seeding, the nanofibrous substrates were sterilized and decontaminated by exposing them to a UV light from a commercially available lamp (Sylvania, 15 W—254 nm) for 30 min. A distance of 45 cm between the lamp and the sample surface and a UV intensity of 300 μW·cm^−2^ were previously revealed to sterilize plasma-treated fibers without harming their morphology nor changing their plasma-induced surface chemistry [53].

PC-12 cells (3.0 × 10^4^ cells/sample) were then seeded onto the nanofibrous scaffolds placed in a 24-well culture plate and incubated at 37 °C in a humidified atmosphere containing 5% CO_2_. To do so, a differentiation medium composed of RPMI 1640 Glutamax supplemented with 1% penicillin/streptomycin, 10% heat-inactivated horse serum and 50 ng/mL human β-nerve growth factor (NGF—PeproTech 450-01) was instead used to stimulate the formation of neurites. 

To access the neurite outgrowth from PC-12 cells, an immunofluorescent staining was conducted. Firstly, the cells (10 days post-seeding) were washed with phosphate-buffered saline (PBS) and fixed with 4% *v*/*v* paraformaldehyde for 20 min at room temperature. The membrane of the fixed cells was then permeabilized by placing the samples in 0.1% Triton X-100 in PBS at room temperature for 45 min. After rinsing the scaffolds with PBS, they were incubated for 1 h in a blocking solution containing 5% *w*/*v* bovine serum albumin (BSA), 0.05% v/v Tween-20 and 0.05% *w*/*v* sodium azide in PBS. The cells were then successively incubated in a primary mouse monoclonal anti-β-tubulin III antibody (Sigma-Aldrich) solution and then in a goat anti-mouse IgG (H+L) highly cross-adsorbed secondary antibody (Alexa Fluor Plus 488, Thermo Fisher Scientific) solution overnight at 4 °C in a humidified chamber each time. Both antibody (1:1000) solutions were prepared in a washing buffer containing 1% BSA and 0.05% Tween-20. Afterwards, the samples were washed with PBS, and the cell nuclei were stained with 4′,6-diamidino-2-phenylindole-dihydrochloride (DAPI, Sigma-Aldrich) for 10 min. Finally, the stained cells were observed and imaged using a fluorescence microscope (Olympus IX81). The density of the formed neurites was assessed from the acquired fluorescent images of each condition making use of the Simple Neurite Tracer plugin of the ImageJ software, which is a semi-automatic plugin to define neurite outgrowth. To do so, the fluorescent images were first converted to binary images. To delineate individual neurites, the starting point and ending point of every neurite path were manually defined so that the path can be afterwards automatically generated and recorded. After delineating all neurites in the image, the neurite density was calculated by dividing the number of the neurites recorded by the Simple Neurite Tracer by the total area of the fluorescent image. A statistical analysis was performed using one-way analysis of variance (ANOVA) with a Tukey post hoc test at a significance level of 0.05.

## 3. Results and Discussion

### 3.1. Physical Characterization of the Polymer Solutions

In this work, different polymeric solutions were subjected to an APPJ treatment prior to the electrospinning process. Such treatment was shown to trigger a degradation of the solvent molecules while preserving the polymeric macromolecules [51]. Therefore, some liquid properties including pH, conductivity and viscosity were expected to change leading to polymeric solutions characterized by an improved electrospinnability. 

**Figure 2:**
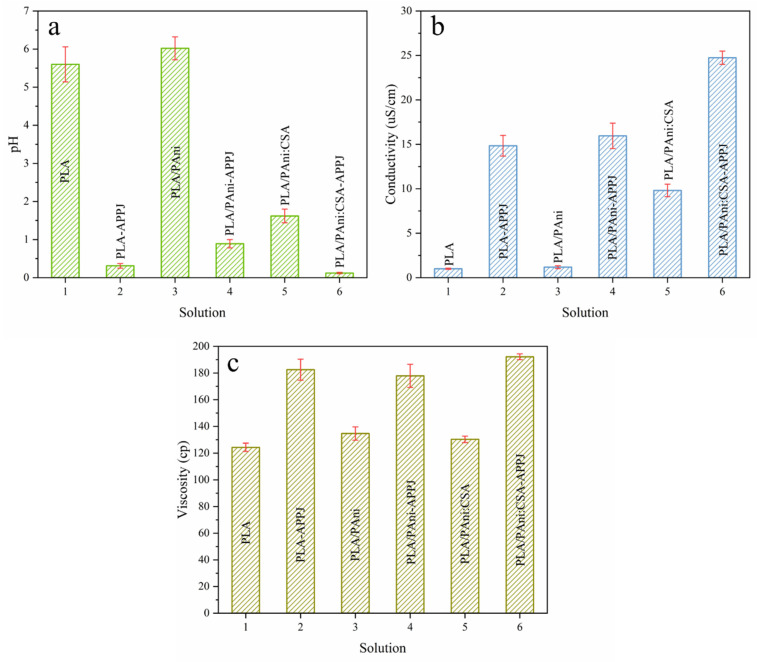
Liquid properties of the different polymer solutions before and after APPJ treatment, (**a**) pH, (**b**) conductivity and (**c**) viscosity.

Figure 2 depicts the bar plots of the pH, conductivity and viscosity of the different polymeric solutions under study, namely PLA, PLA/PAni and PLA/PAni:CSA before and after APPJ treatment. Results revealed that, for all solution groups, the conductivity and viscosity, which constitute two of the key properties affecting the electrospinning process and the ensuing nanofiber morphology, were largely increased. In this context, it is worth mentioning that during the APPJ treatment, an evaporation of a significant volume of the solvents occurred thereby leading to an enhanced polymer concentration and in turn to the observed amplification in the solution viscosity. Nonetheless, this solvent loss was not the only reason behind the enhanced viscosity as previously confirmed in comparative studies between plasma-treated solutions and control solutions having the same final concentration [54]. In fact, upon the plasma-induced degradation of CHCl_3_ and DMF molecules, some chemical species characterized by high conductivities, such as hydrochloride acid (HCl) and HNO_3,_ were probably formed [47], which could explain the enhanced solution conductivity. Such species are additionally highly polar which may lead to a higher PLA or PLA/PAni solubility and thus to the expansion of the polymers coils leading to a further enhancement in the solution viscosity. The process behind the formation of HCl is explained in detail in Section 3.2. Another newly formulated hypothesis, based on the intermolecular hydrodynamic interactions and/or entanglements between polymeric chains that determine the polymer solution viscosity, was brought out in a previous work of our research group. The viscosity of the untreated solutions actually fits into a modestly concentrated regime where the polymeric chains are not fully separated by solvent molecules but start initiating entanglements or contact between them. The APPJ treatment probably engendered the generation of some non-neutral species, such as Cl^-^ and HCOO^-^, which charged the polymeric chains. As a consequence of this charging, the hydrodynamic volume of the polymeric macromolecules was likely to be amplified, thus promoting more interactions between the polymeric chains and the solvent molecules and between the different polymeric chains. These enhanced interactions could potentially trigger an additional increase in the viscosity of the plasma-treated solutions [55]. 

**Figure 3:**
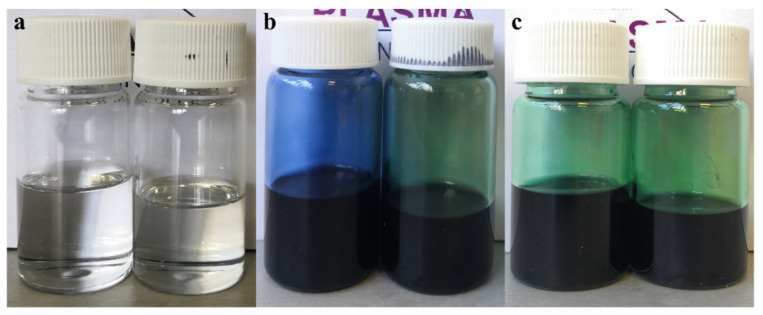
Photographs of the polymer solutions pre- and post-plasma treatment: (**a**) PLA (left) and PLA-APPJ (right), (**b**) PLA/PAni (left) and PLA/PAni-APPJ (right) and (**c**) PLA/PAni:CSA (left) and PLA/PAni:CSA-APPJ (right).

Unlike the detected variations in these two previously described properties, the plasma-induced pH change of the different solution groups is relatively less straightforward. As seen in Figure 2a, among all untreated solutions, PLA/PAni:CSA had a lower pH comparing with PLA and PLA/PAni, which can be attributed to the presence of the organic acid. After the APPJ treatment, all pH values of the different groups declined, which is again due to the plasma-induced generation of some acids, such as HCl originating from the degradation of chloroform being one of the used solvents [47]. However, after the APPJ treatment, the pH reduction of the PLA/PAni solution was notably less prominent than in case of the other groups. When taking a look at the photographs of the different polymer solutions shown in Figure 3, one can notice a clear color transition of the PLA/PAni solution from blue to green post-treatment. According to literature, PAni EB is blue, while the PAni ES (acid-doped form) is green [22]. The observed color transition suggests that a doping behavior of PAni EB to salt occurred during the APPJ treatment. The mechanism of this doping will be further analyzed in Section 3.2. Given the fact that the doping/dedoping of PAni is an acid-base chemistry process, one can assume that the observed doping behavior could consume some APPJ-generated acids. Consequently, a reduction in the concentration of hydrogen ions befell, which can explain the smaller decrease in the pH value of the PLA/PAni solution.

### 3.2. Morphology of the Electrospun Nanofibrous Scaffolds 

**Figure 4:**
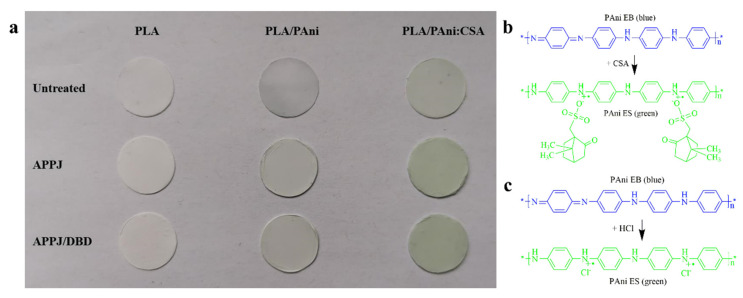
(**a**) Photographs of untreated, APPJ-treated and APPJ/DBD-treated PLA (left column), PLA/PAni (middle column), PLA/PAni:CSA (right column) nanofibrous scaffolds. Schematic representation of PAni EB doped with the organic acid CSA (**b**) and the inorganic acid HCl (**c**) generated in the APPJ treatment.

After the preparation of the different polymeric solutions, three types of nanofibrous scaffolds were electrospun, namely PLA, PLA/PAni and PLA/PAni:CSA. As can be visualized from the photographs of the obtained mats (Figure 4a), the pure PLA nanofibers were white in color. The presence of PAni in the composite nanofibers triggered variable color changes depending on its doping state. When a small fraction of PAni was blended with PLA, the composite PLA/PAni nanofibers turned into a light blue color given the fact that PAni EB is blue. For PLA/PAni:CSA nanofibers, the addition of the organic acid could dope PAni from its EB form to its ES form, making the scaffold light green-colored. This doping process of PAni EB with CSA is represented in Figure 4b. The colors of the different mats were quite in accordance with the colors of the corresponding polymer solutions as detected in Figure 3. 

When APPJ treatments were applied to the different polymer solutions, the resultant nanofibrous scaffolds showed different (shades of) colors. PLA mats electrospun from the APPJ-modified solution exhibited a whiter color when compared to the untreated sample. The same change illustrated by a darker shade of green is also observed in case of the PLA/PAni:CSA nanofibers originating from the corresponding APPJ-modified solution compared to the ones originating from the untreated solution. As already mentioned, the APPJ treatment can significantly improve the electrospinnability of polymeric solutions [46,51], giving rise to more uniform and denser nanofiber morphology (as will be shown in the following paragraph). Moreover, our research group has recently demonstrated that the treatment could enhance the nanofibers deposition yield, leading to thicker mats, which implies a visual further accentuation of the color shade of the mats [55]. Interestingly, PLA/PAni nanofibers showed an obvious color transition from light blue to light green when electrospun from their corresponding APPJ-modified solution. This observation follows the same color transition observed for the untreated and the plasma-treated solutions (Figure 3b). This transition can be attributed to the fact that a portion of PAni EB was doped upon exposure to the argon plasma jet. In our previous study, it was found that HCl was generated by plasma when using chloroform as one of the solvents. This process was carefully analyzed and described in literature based on Equation (1) [47]. Briefly speaking, the interactions between chloroform and the electrons present in the argon plasma induce chloroform decomposition, which is responsible for the dissociation of C–Cl bonds. The generated ·Cl can further react with chloroform, leading to the generation of HCl. In the case of the plasma-treated PLA/PAni solution, the generated HCl can subsequently induce the doping of PAni EB to PAni ES as shown in Figure 4c, which accounts for the color transition mentioned above.

In a next step, the APPJ-treated nanofiber scaffolds were further treated post-electrospinning with a DBD sustained in nitrogen. Based on a visual macroscopic observation, the color of these mats was not further affected by the DBD treatment (Figure 4a).

(1) $${\mathrm{CHCl}}_{3}+{\;e}^{-}\;\to \;\cdot \mathrm{Cl}\;+\cdot {\mathrm{CHCl}}_{2}$$

(2) $$\cdot \mathrm{Cl}\;+{\;\mathrm{CHCl}}_{3}\;\to \;\cdot {\mathrm{CCl}}_{3}+\mathrm{HCl}$$

**Figure 5:**
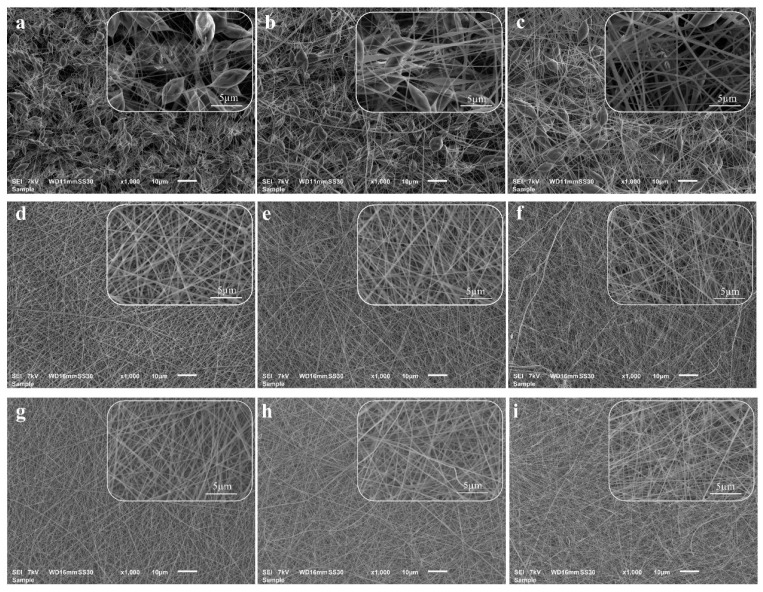
SEM images of (**a**) PLA, (**b**) PLA/PAni and (**c**) PLA/PAni:CSA nanofibers electrospun from their corresponding untreated solutions; (**d**) PLA, (**e**) PLA/PAni, (**f**) PLA/PAni:CSA nanofibers electrospun from their corresponding APPJ-modified solutions; (**g**) PLA, (**h**) PLA/PAni, (**i**) PLA/PAni:CSA electrospun nanofibers after APPJ/DBD treatments (DBD exposure time: 15 s—Scale bar: large images 10 µm and inset images 5 µm).

To further investigate the effect of the pre- and post-electrospinning plasma treatments on the morphology of the electrospun nanofibers, SEM images of the samples were acquired and compared. Figure 5a–c shows PLA, PLA/PAni, and PLA/PAni:CSA nanofibers electrospun from their corresponding untreated solutions. The nanofibers of all groups showed an inhomogeneous morphology interrupted by the occurrence of numerous beads instead of being nicely elongated. The beads exhibited a spindle-like shape with a maximal length of around 10 um and an average width of around several microns, indicating a very poor electrospinnability. This mediocre morphology is mainly caused by the relative low conductivities and viscosities of the untreated solutions as seen in Figure 2. In fact, when these physical properties are low, the electrical field applied during electrospinning drives the polymer jet to break into spherical or spindle-like fragments in order to minimize the surface energy. During this process, the surface tension of the jet causes a radial contraction that ends up generating beads [51,55]. When taking a closer look at the SEM images of all untreated mats, one can notice a lower number of beads on the PLA/PAni:CSA nanofibers compared to the other two groups. The enhanced conductivity associated with the presence of CSA in the corresponding solution is presumably leading to an enhanced electrical stretching of the polymer jet during electrospinning which counteracted its radial contraction, thus reducing the formation of beads. 

When the pre-electrospinning APPJ treatment was conducted, the morphology of the nanofibers was largely changed. Figure 5d–f depicts the SEM images of PLA, PLA/PAni and PLA/PAni:CSA nanofibers electrospun from their corresponding APPJ-modified solutions. A largely improved electrospinnability illustrated by uniform, smooth, elongated and bead-less nanofibers was obtained for all groups. This notable improvement is attributable to the plasma-induced increase in the conductivity and viscosity that could respectively improve the electrical stretching [56] and entanglement [57] of the polymer chains during the electrospinning process, thus completely overcoming the breakage of the jet into fragments before reaching the collector.

In contrast to the APPJ treatment, the post-electrospinning DBD treatment of the electrospun mats exhibited no visible influence on the nanofibrous morphology as can be seen in Figure 5g–i. Moreover, no statistical difference in the nanofiber diameters was detected before and after the treatment suggesting that the N_2_ plasma did not cause any melting or etching effects and as such did not trigger any physical damage to the delicate nanofibrous structure (Table 1). In this context, it is worth mentioning that because of the occurrence of massive beads, the diameters of the nanofibers electrospun from the untreated solutions were unmeasurable and are as such not presented in Table 1. Regardless of the plasma treatment, when comparing the average fiber diameters of the different nanofibers mats, one can notice lower values for PLA/PAni:CSA nanofibers (170.0 ± 26.9 nm post-APPJ and 175.3 ± 40.1 nm post-APPJ/DBD). This can be again attributed to the presence of CSA in the solution, which improved its conductivity and as such resulted in a more pronounced elongation of the polymer jet during electrospinning, which in turn led to the deposition of thinner fibers. 

### 3.3. Wettability of Nanofiber Scaffolds

Before discussing the surface wettability of the electrospun mats, it is important to note that most biopolymers including PLA are intrinsically hydrophobic materials [58]. When such materials are further processed into the form of 3D nanofibrous scaffolds, amplified WCAs are measured on their surface compared to their 2D counterparts. This increased WCA is due to the presence of inter-fibrous pores in which the surrounding air gets entrapped, thus hampering the infiltration and spreading of water [31,59]. In this work, two plasma treatments (APPJ and DBD) are conducted, and it is interesting to explore how the two applied plasma treatments could affect the wettability of the scaffolds. 

When measuring the WCAs of the untreated PLA, PLA/PAni and PLA/PAni:CSA nanofibrous scaffolds, high values of 141.9°, 137.1° and 138.6° were, respectively, obtained which corroborated the previously described high hydrophobicity of PLA-based nanofibers (Table 2, the representative WCA images can be found in Figure S1). An approximate decrease of 10° was then detected for the 3 nanofibrous groups when they were fabricated from their APPJ-modified solutions. This slight reduction is the WCA values was also previously spotted on PCL nanofibers electrospun from an APPJ-modified solution compared to their counterparts fabricated from an untreated solution [60]. Such a minor increase in the surface wettability can be presumably attributed to two reasons. On the one hand, the more uniform morphology of the nanofibers and the elimination of their beaded structure that resulted from the APPJ treatment was likely to promote the spreading of the water drops leading to reduced angles. On the other hand, the APPJ-induced minor changes in the elemental composition of the nanofibers, which will be further revealed in the next section, making use of XPS data, may also, to some extent, affect their wettability. Despite these changes, it is worth mentioning that all APPJ-treated samples were still highly hydrophobic (WCA > 120°). Overall, it was substantiated that the APPJ treatment, being a liquid modification technique of polymeric solutions, does not significantly affect the surface wettability of the ensuing electrospun nanofiber scaffolds. 

**Figure 6:**
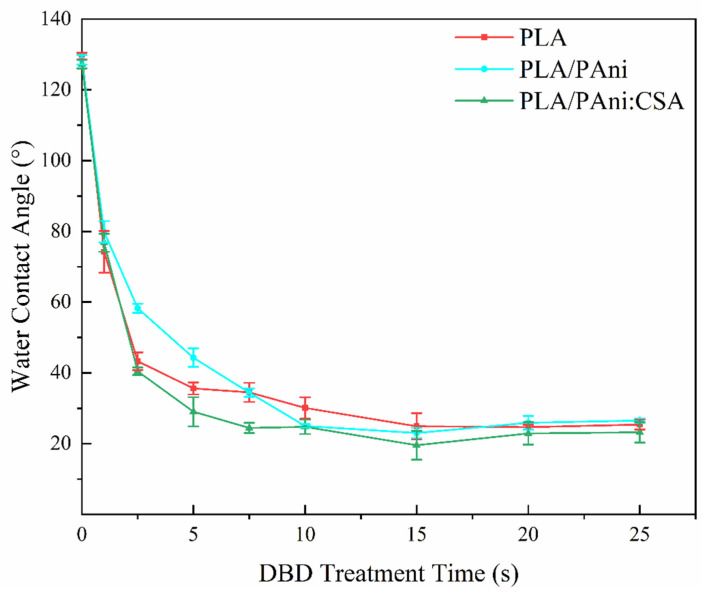
Evolution of the WCA values as a function of nitrogen DBD treatment time.

In contrast to the APPJ treatment of polymeric solutions, the DBD treatment of polymeric substrates is a well-established strategy that efficiently enhances their surface wettability [58]. As such, the hydrophobic nanofibrous scaffolds electrospun from the APPJ-modified solutions were subjected to DBD treatments sustained in N_2_. The evolution of their WCA values as a function of the treatment time is shown in Figure 6, and the representative WCA images of PLA, PLA/PAni and PLA/PAni:CSA scaffolds after 15 s DBD treatment can be found in Figure S1. Results revealed that for very short exposure times to the N_2_ DBD, a prompt switch from hydrophobic to hydrophilic surface states occurred for all the samples. In particular, after a plasma treatment of only 2.5 s, the WCA of PLA, PLA/PAni and PLA/PAni:CSA scaffolds decreased from 129.5° to 43.3°, from 128.5° to 58.3° and from 127.2° to 40.4°, respectively. Such rapid wettability alterations are consistent with the results of a previous study in which PCL nanofibers were also subjected to a medium-pressure DBD treatment [31]. This is due to the plasma-induced incorporation of polar groups on the surface of the nanofibers as will be discussed in the following section. In fact, when a certain chemical hydrophilicity is reached on the surface of nanofibers, the deposited water drop of low surface tension starts to overpower the air entrapment by its inability to be further held on the surface. This leads to its infiltration inside the fibrous mesh, which explains the sudden big drop in surface wettability. As the treatment time increases, a more gradual decrease in the WCA can be perceived which is attributable to more polar groups being grafted on the surface. Finally, after 15 s of plasma exposure, PLA, PLA/PAni and PLA/PAni:CSA nanofibrous scaffolds reached their saturated wettability state with WCA values of 24.9°, 23.0° and 19.6°, respectively. This suggests that a further extension of the treatment time, within certain limits, would not elicit any additional surface chemical modifications. 

According to the performed WCA analysis, a DBD exposure of 15 s was selected as optimal treatment time given the fact that it marked a saturation of the treatment effect for all 3 nanofibrous groups. In what follows, untreated, APPJ-treated and DBD-treated samples for solely this optimal time (15 s) will be subjected to XPS and cell test analyses. 

### 3.4. Surface Chemical Characterization of the Nanofibers

To investigate the surface chemical composition changes before and after the two applied plasma treatments, the untreated and plasma-treated samples were subjected to an XPS analysis. Compared with the straightforward analysis of the pure PLA nanofibers, the analysis of PLA/PAni and PLA/PAni:CSA composites is egregiously complex. This is due to the fact that the latter two materials are binary and ternary systems that were further exposed to different plasma species in each of the applied pre- and post-electrospinning plasma treatments. Moreover, the XPS results of the latter two conditions are not expected to be significantly different from the pure PLA samples when considering the detection limits of XPS, since the concentration of PLA was dominant in the PLA/PAni and PLA/PAni:CSA composites. Therefore, to better reveal the effects of the two applied plasma treatments, the XPS results of PLA samples were only presented. 

**Figure 7:**
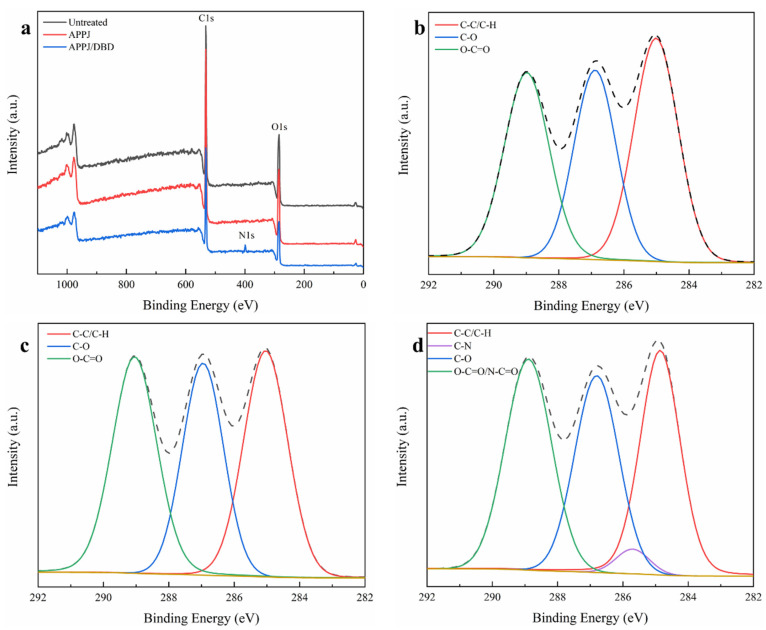
(**a**) Survey scan spectra of untreated, APPJ-treated and APPJ/DBD-treated PLA nanofibers; examples of high resolution C1*s* curve fitting of untreated (**b**), APPJ-treated (**c**) and APPJ/DBD-treated (**d**) PLA nanofibers.

Table 3 encompasses the elemental composition results of the different PLA nanofibers, as determined from their survey spectra shown in Figure 7a. Results revealed that the surface of the untreated PLA nanofibers exhibited a carbon content of 61.2% and an oxygen content of 38.8%, which is in line with the theoretical values (60% of carbon and 40% of oxygen) deduced from the PLA molecular structure. After APPJ treatment of the polymer solutions, a slight increase in the oxygen content reaching 39.6% and an accompanying decrease in carbon content reaching 60.4% were detected on the surface of the nanofibers. Although minor, this increase in oxygen content was recurrently perceived in literature when for instance PCL, PLA and Polyactive^®^ nanofibers were electrospun from plasma-treated solutions [46,60,61]. The APPJ-induced incorporation of oxygen may occur when residual H_2_O or O_2_ impurities in Ar or originating from the surrounding ambient air interact with different plasma particles forming reactive species, such as hydroxyl (OH) and atomic oxygen, which in turn interact with the polymer chains [50]. Moreover, this surface incorporation of polar oxygen-containing groups can be, to a small extent, linked back to the slightly decreased WCA values of the nanofibrous scaffolds after APPJ treatment. When PLA nanofibers electrospun from APPJ-modified solutions were further plasma treated making use of the N_2_ DBD, 3.3% of nitrogen was found to be introduced on their surface. However, different from what was observed in some literature, where oxygen was additionally implanted on the substrate’s surface after N_2_ plasma treatment [62,63,64], the O/C ratio under study was found not to significantly change before (0.656) and after (0.659) treatment. This result, suggesting that oxygen was not efficiently grafted on the surface, is in agreement with our previous study in which a N_2_ DBD treatment was performed on PLA films [33]. Overall, both the APPJ treatment of the polymer solution and the DBD treatment of the electrospun nanofibers could successfully introduce new atoms: the APPJ treatment introduced a small amount of oxygen on the nanofibers, while the DBD treatment mainly induced a surface nitrogen incorporation.

**Figure 8:**
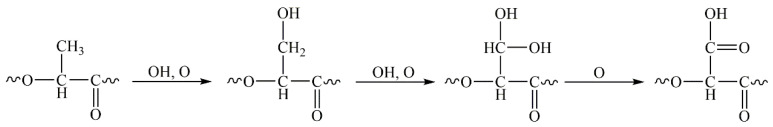
Proposed mechanism for the APPJ treatment of PLA solutions in argon plasma.

To better understand the chemical changes induced by the applied two plasma treatments, high-resolution C1*s* spectra of untreated, APPJ-treated and APPJ/DBD-treated PLA nanofibers were curve fitted as shown in Figure 7b–d. This procedure was performed to reveal what types of functional groups were specifically incorporated upon both treatments. According to the molecular structure of PLA and to literature, the C1*s* curve of the untreated sample was decomposed into three peaks: a peak at 285.0 eV corresponding to C–C/C–H, a peak at 286.8 eV assigned to C–O and a peak at 289.0 eV representing O–C=O bonds [33]. As shown in Table 4, the relative concentrations of these three peaks were 37.6%, 30.0% and 32.4%, respectively. After the implementation of the APPJ treatment, a slight increase in the relative concentration of O–C=O bonds reaching a value of 34.6% was spotted. In turn, an accompanying slight decrease in the concentration of C–C/C–H bonds to a value of 35.2% was detected with an unchanged C–O bond content. The results are in close agreement with the variation in the elemental composition determined by the survey scans and came to corroborate the significance of the small oxygen increase on the surface. Similar results also revealing a slight relative increase in O–C=O bond content were previously obtained when treating PLA solutions with an APPJ [46]. However, the APPJ treatment of PCL solutions was shown to mainly contribute to an increase in the C–O bond content rather than the O–C=O bonds [51]. This difference in bond formation may be attributed to the initially different molecular structures of PLA and PCL, i.e., carboxyl (O–C=O) functionalities were more readily formed when the reactive species in the APPJ plasma interacted with PLA molecules. Figure 8 depicts a proposed mechanism for this process. In a first step, the C–H bonds of methyl (-CH_3_) groups turn into alcohol (C–OH) groups via the substitution of a hydrogen atom with an oxygen atom (O) or hydroxyl group (OH). A second alcohol group is then formed on the same carbon atom when the polymer chains are further exposed to the plasma jet, leading to the formation of a geminal 1,1-diol (R–C–(OH)_2_) [65]. This hypothesis can be supported by the fact that the main excited species in the plasma were atomic oxygen and hydroxyl radicals as could be detected by recording in situ the optical emission spectrum of the APPJ (shown in Figure S2). Lastly, on the basis of the above-stated reactions, the unstable geminal diol can be easily converted to a carboxylic acid through dehydrogenation when strong oxidants such as OH radicals and atomic O exist [66]. This hypothesis is presumably accounting for the decreased concentration of C–C/C–H bonds and increased concentration of O–C=O bonds after APPJ treatment.

In the case of the DBD sustained in N_2_, the dominant reactive species are probably atomic nitrogen radicals (N^·^). This radical can be efficiently generated by the recombination of $N_{2}^{+}$ ions with electrons at the surface of the nanofibers ($N_{2}^{+}$ + $e_{surface}^{-}$ → 2N^·^ + 4eV). Concurrently, the energy released by this exothermic reaction is high enough to break the chemical bonds of PLA [67]. Additionally, the non-reactive species in plasma, for example, the photons and excited molecular N_2_^*^, can also break the C–C or C–H bonds, creating surface radicals. The created polymer radicals can then react with reactive species (N^·^ in this work), which results in the incorporation of nitrogen-containing functional groups onto the surface of the nanofibers. In theory, an N_2_ DBD can create a variety of functional groups including amines (NH_2_, NH), imines (C=N) and amides (O=C–N) [67]. However, the functional groups created in a certain work usually involve one or part of the above-listed groups, as their formation depends on the polymer molecular structure and the applied plasma conditions. To distinguish the distinct functional groups created in this work, high-resolution N1*s* spectra of APPJ/DBD-treated PLA were curve-fitted as shown in Figure S3. It should be noted that the positioning of similar nitrogen-bonded component peaks within the N1s envelope shows a lot of inconsistencies in literature, i.e., one can observe some discrepancies in binding energies in different publications [68]. The binding energies adopted in this work were the most consistently reported in literature: primary amine, secondary amine, amide and charged amine peaks were positioned at 398.7 eV, 400.0 eV, 400.9 eV and 401.6 eV, respectively [63,64] (Table S1). From the deconvolution of the N1*s* spectra, it was found that the N_2_ DBD majorly contributed to the incorporation of secondary amines (68.3%) and amides (16.2%), with a minor implantation of primary amines (7.4%) and charged amines (8.0%). 

Figure 7d shows the C1*s* peak of PLA nanofibers after N_2_ DBD treatment. Besides the three peaks used in the curve fitting of the untreated and APPJ-treated samples, a peak at 285.7 eV assigned to C–N bonds was added. Moreover, the peak at 289.0 eV is redefined to O–C=O and N–C=O, as the binding energy difference between both groups is too small to allow a separation within acceptable error bars. The addition of a C–N peak led to a good fitting of the C1*s* spectrum with small chi-squared errors (0.84 ± 0.36). Additionally, the obtained results were quite consistent with what was observed in the N1*s* curve fitting. In fact, the DBD treatment was responsible for the incorporation of 3.0% of C–N bonds at the expense of C–C/C–H bonds for which the relative concentration was found to decrease from 35.2% (APPJ-treated counterpart) to 33.1%. The peak at 289.0 eV also seems to be slightly decreased despite the newly created N–C=O groups. This unusual behavior could be most likely interpreted by the fact that the incorporation of N–C=O groups mainly occurred through the substitution of the oxygen atom in O–C=O groups by a nitrogen. Therefore, the total concentration of the groups at 289.0 eV was not increased when exposed to the DBD treatment. These results, together with the results deduced from the survey scan analysis, confirm that nitrogen-containing groups were incorporated on the surface of PLA nanofibers which could again be linked back to the noticeable decrease in the WCA values seen in Section 3.3 given the high polarity of these groups.

### 3.5. Cell-Scaffold Interactions: In Vitro PC-12 Cell Study

As shown above, the pre-electrospinning APPJ treatment of the polymeric solutions and the post-electrospinning DBD treatment of the ensuing nanofibers led to different changes in the final scaffold properties. The former plasma treatment contributed to a notable transformation in the fiber morphology from a mediocre bead-containing configuration to a uniform and almost bead-free one. The latter plasma treatment could incorporate nitrogen-containing functionalities, thus efficiently enhancing the surface hydrophilicity of the nanofibers. In order to access the influence of these beneficial plasma-induced changes on the behavior of electro-sensitive cells, a comparative in vitro study implemented on untreated, APPJ-treated and APPJ/DBD-treated samples was performed using PC-12 cells. This cell type was chosen given its ability to undergo a neuronal differentiation as a response to an exposure to NGF. The neuron-like differentiated cells are electrically excitable and are able to form and extend branched neurites in adequate stimulating microenvironments [69]. As such, this in vitro test using this specific cell type constitutes an accurate primary proof-of-concept study predicting the potential of the developed (non-)conducting based scaffolds in nerve TE.

**Figure 9:**
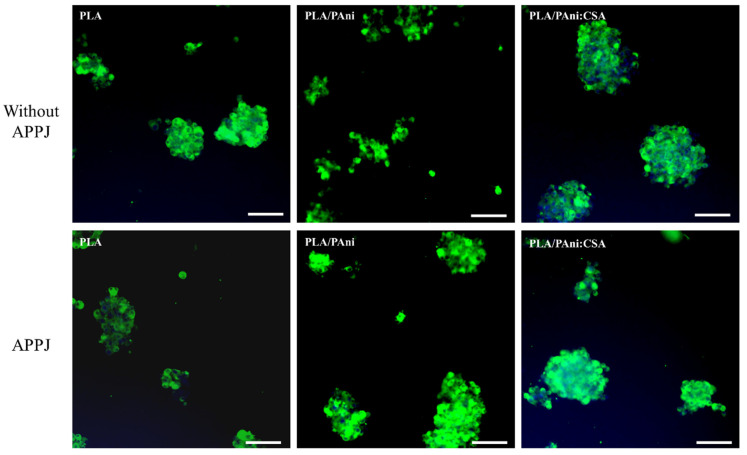
Representative immunofluorescent images of PC-12 cells cultured on untreated and APPJ-treated PLA, PLA/PAni and PLA/PAni:CSA nanofibers for 10 days (Scale bar: 100 µm).

To clearly assess the effects of the two treatments separately, the tests were carried out in two steps. In the first step, PC-12 cells were cultured on the untreated and APPJ-treated nanofibers. It should be noted that all samples included in this step were hydrophobic as they did not undergo any DBD surface modification. Figure 9 displays the representative fluorescent images after 10 days of culturing. Before deeply examining the ability of the cells to differentiate and extend neurites, it is important to assess the initial primordial cell affinity (i.e., adhesion) towards the surface. A low cell density was perceived on all samples regardless of whether the APPJ was performed or not. A high tendency of cell agglomerations leading to (big) cell clusters which is a sign of mediocre cell adhesion/affinity towards the nanofibers was observed. This cell clustering might elicit the death of the cells present in the inner deep region of the cluster as a consequence of insufficient nutrient supply to the central areas. Despite the fact that the morphology of the nanofibers was largely improved, post-APPJ treatment in terms of uniformity and bead occurrence, similar cell densities and morphologies were detected on both untreated and treated samples. 

**Figure 10:**
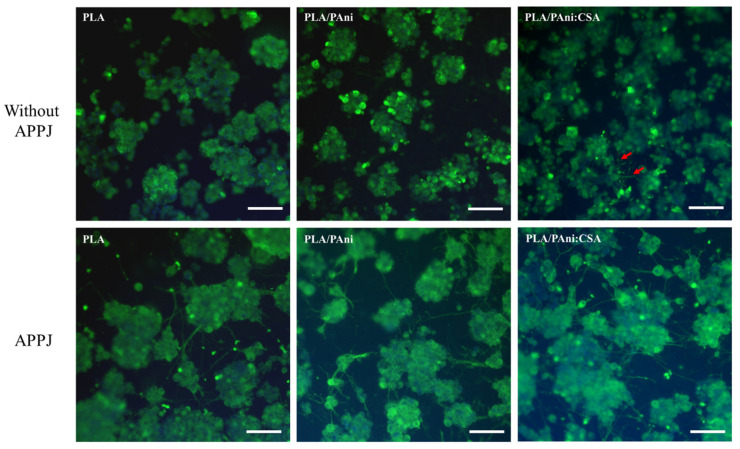
Representative immunofluorescent images of PC-12 cells cultured on DBD-treated and APPJ/DBD-treated PLA, PLA/PAni and PLA/PAni:CSA nanofibers for 10 days (Scale bar: 100 µm).

In a second step, all samples were subjected to the optimized N_2_ DBD treatment before cell seeding, i.e., all surfaces were hydrophilic. Figure 10 shows the representative fluorescent images of PC-12 cells 10 days after seeding. Compared with the results obtained in the first step, a significantly enhanced cell density and a more homogeneously dispersed cell distribution over the whole substrate surface could be visualized in all conditions. These noticeably improved PC-12 cell-nanofibers interactions (adhesion and dispersion) can be correlated to the DBD-induced alteration of the surface chemistry illustrated by the implantation of polar nitrogen-containing groups and the consequent enhanced surface wettability. In fact, an improved hydrophilicity elicits the adsorption of more proteins onto the scaffolds’ surface. PC-12 cell receptors, mainly the integrin family receptors, can subsequently bind to the adsorbed proteins triggering the generation of numerous focal adhesion sites promoting: (1) a tighter attachment of individual cells on the surface and (2) the adhesion of more cells on the wettable surface [70]. In contrast, the untreated surfaces exhibited a lower content of polar groups (absence of plasma-induced nitrogen-containing functionalities) that can act as protein binding sites and as such cell binding sites in the initial stages of cell attachment. This explains the lower cell density on the untreated hydrophobic surfaces and the higher cell tendency to form clusters rather than attaching to the nanofibers’ surface. Wang et al. have also previously detected a significantly improved PC-12 cell adhesion on plasma-treated poly(lactic-co-glycolic acid) nanofibers compared to untreated nanofibers [35]. These results suggest that, compared with the substrate morphology, the surface wettability is a considerably more influential property in determining the initial PC-12 cell-material adhesion and uniform dispersion.

Besides the initial cell affinity towards the surface, the outgrowth of neurites underlying the neuronal differentiation of PC-12 cells is another important characterization to be taken into account. The cells cultured on the hydrophobic nanofibers (no DBD treatment) displayed in Figure 9 did not show any sign of neurite extension. This is most probably due to the initial poor cell adhesion hampering any further cell differentiation. In contrast, the hydrophilic APPJ/DBD-treated nanofibers displayed in the bottom row of Figure 10 show some conspicuously outgrown neurites, which are able to connect PC-12 cell clusters to form a network. However, neurite outgrowth was barely observed on the samples only exposed to the DBD and not to the APPJ (top row of Figure 10) exhibiting poor nanofibers’ morphology. A very small number of short neurites (marked by red arrows) could only be observed in this case on the PLA/PAni:CSA nanofibers. To further confirm this observation, more fluorescent images of lower magnification taken from the different DBD-treated fiber conditions are displayed in Figure S4. The greatly improved neurite extension on the APPJ/DBD-treated nanofibers compared to the DBD-treated nanofibers, which can be clearly visualized in the bottom row of Figure 10 and which is corroborated in Figure S4, undoubtedly indicates the beneficial effect of the APPJ treatment in enhancing PC-12 differentiation. As mentioned above, the APPJ treatment did not contribute to significant changes in the surface chemical composition nor in the surface wettability, but hugely improved the morphological nanofibers’ uniformity and hampered the bead formation. Neurite outgrowth from PC-12 cells is actually known to be regulated by the geometrical features of the underlying substrates on which they are cultured [11,71,72]. Hence, one can conclude that neurite extension on the substrates only exposed to the DBD was largely halted by the wide-distributed beads forming obstacles impeding its initiation and disrupting the elongation of the few outgrown neurites, thus accounting for the low density of short neurites. 

In order to further compare the neurite outgrowth on APPJ/DBD-treated PLA, PLA/PAni and PLA/PAni:CSA nanofibers, the neurite density was quantified, and the results are presented in Figure S5. A significantly (ANOVA, *p* = 0.014) higher neurite density was detected on PLA/PAni:CSA nanofibers (6.7 × 10^3^ /cm^2^) compared to PLA scaffolds (4.6 × 10^3^ /cm^2^). Nonetheless, when PLA was blended with the non-conducting PAni EB, the neurite density (4.8 × 10^3^ /cm^2^) was almost similar to PLA scaffolds (ANOVA, *p* = 0.934). These results suggest that the presence of the conducting form ES of PAni (PAni:CSA) could positively affect and further stimulate neurite outgrowth. Some similar findings, illustrated by the improvement of the performances of electro-sensitive cells/tissues upon the presence of conductive PAni, were also reported in previous studies. For instance, the addition of conductive PAni in collagen favored the attachment and contraction of rat cardiomyocytes [8]; the in situ polymerization of PAni on a cellulose hydrogel promoted the sciatic nerve regeneration of adult rats [15]; and the addition of conductive PAni to PCL nanofibers enhanced the differentiation of PC-12 cells [16]. It should be noted that these studies, together with our work, were all conducted without external electrical stimulation, indicating that the sole presence of conductive PAni without any combined external stimulus can also promote electrical-sensitive cell behaviors. Despite the fact that the APPJ treatment of the PLA/PAni solutions could lead to a certain level of doping of PAni as was illustrated by a color transition from blue to green, the neurite density was not significantly enhanced compared to PLA scaffolds. This suggests that the probable doping effect was not sufficient to reach the same conductivity level of the PLA/PAni:CSA scaffolds. The interactions of the non-conductive form of PAni (PAni EB) with cells were not reported in the above-cited studies. However, from the present work, it can be concluded that the presence of a non/semi-conducting form of PAni is not as effective as its conducting form in promoting neurite outgrowth.

In summary, the pre- and post-electrospinning plasma treatments could synergistically enhance the performances of PC-12 cells as follows: (1) the DBD treatment promoted the surface wettability of the nanofibers resulting in improved cell adhesion and dispersion and (2) the APPJ treatment largely eliminated the beaded structures, favoring the extension of neurites and the formation of a neuronal network between PC-12 cell clusters. Lastly, a larger number of neurites was perceived on PLA/PAni:CSA scaffolds, indicating that the addition of conducting polymers can also promote neurite outgrowth without any external electrical stimulation. 

## 4. Conclusions

With the aim of developing refined electrospun scaffolds for nerve TE applications, a combinatorial strategy merging exclusive pre- and post-electrospinning plasma treatments of blended biopolymer/conducting polymer was adopted. Three groups of nanofiber scaffolds, namely PLA, PLA/PAni and PLA/PAni:CSA, were considered for an extensive comparative analysis. Firstly, an APPJ treatment of the corresponding polymeric solutions was conducted before electrospinning to improve their electrospinnability. A characterization of the physical properties of all solutions revealed an enhanced viscosity, conductivity and reduced pH post-treatment. These beneficial changes led to a significantly enhanced electrospinnability illustrated by highly improved nanofibers’ morphology in terms of uniformity and elimination of beads. After electrospinning, a second N_2_-sustained DBD treatment of the electrospun nanofibrous scaffolds was carried out to enhance their surface chemical properties for better cell-material interactions. A notably boosted surface wettability, represented by decreased WCAs, of the DBD-treated nanofibers was perceived. This was attributed to the incorporation of 3.3% of nitrogen under the form of C–N and N–C=O bonds on the scaffolds’ surface as revealed by XPS analyses. Finally, as an initial proof-of-concept study for the use of the conceived scaffolds in nerve TE applications, PC-12 cells were chosen to be cultured on the scaffolds. Immunofluorescent images revealed that the DBD treatment greatly improved the cell affinity towards the surface as shown by a higher density of adhered cells and more uniform cell dispersion over the whole nanofibrous mesh. Interestingly, in addition to the necessary DBD treatment, the APPJ treatment of the polymeric solutions, resulting in an improved nanofiber morphology, combined with the DBD treatment led to the extension of a considerably higher number of longer neurites. In fact, the uniform nicely elongated fiber morphology favored the formation of a neuronal network between PC-12 cell clusters while the beads present in untreated samples formed obstacles impeding neurite extension and elongation. In addition, the presence of conducting PAni in the scaffolds further promoted the behavior of PC-12 cells as illustrated by a higher neurite density without any external electrical stimulation. As such, this work presents an exclusive strategy combining different plasma-assisted biofabrication techniques of a blend of biopolymers with conducting polymers as a promising approach in nerve regeneration. Overall, this paper constitutes a reference for the fabrication of more appropriate scaffolds to be used in electro-sensitive TE fields in general.

## Figures and Tables

**Table 1:** Average diameters (nm) of PLA, PLA/PAni and PLA/PAni:CSA nanofibers.

Condition	PLA	PLA/PAni	PLA/PAni:CSA
APPJ	231.5 ± 48.4	217.7 ± 51.2	170.0 ± 26.9
APPJ/DBD	217.5 ± 36.9	217.9 ± 40.3	175.3 ± 40.1

**Table 2:** Water contact angles (°) of untreated and APPJ-treated nanofibrous scaffolds.

Condition	PLA	PLA/PAni	PLA/PAni:CSA
Untreated	141.9 ± 2.8	137.1 ± 1.0	138.6 ± 0.7
APPJ	129.5 ± 1.0	128.5 ± 1.4	127.2 ± 1.2

**Table 3:** Surface elemental composition of untreated, APPJ-treated and APPJ/DBD-treated PLA nanofibers.

Condition	C (at%)	O (at%)	N (at%)	O/C	(O+N)/C
Untreated	61.2 ± 0.5	38.8 ± 0.5	0	0.634	0.634
APPJ	60.4 ± 0.4	39.6 ± 0.4	0	0.656	0.656
APPJ/DBD	58.3 ± 0.4	38.4 ± 0.6	3.3 ± 0.6	0.659	0.715

**Table 4:** Relative concentrations (%) of carbon-containing functional groups on the surface of untreated, APPJ-treated and APPJ/DBD-treated PLA nanofibers.

Condition	C–C/C–H (285.0 eV)	C–N (285.7 eV)	C–O (286.8 eV)	O–C=O/N–C=O (289.0 eV)
Untreated	37.6 ± 0.6	0	30.0 ± 0.6	32.4 ± 0.4
APPJ	35.2 ± 0.6	0	30.2 ± 0.5	34.6 ± 0.4
APPJ/DBD	33.1 ± 0.5	3.0 ± 1.4	30.2 ± 1.1	33.7 ± 0.6

## Data Availability

Data is contained within the article or supplementary material.

## Supplementary Materials

The following supporting information can be downloaded at: https://www.mdpi.com/article/10.3390/polym15010072/s1, Figure S1: Representative WCA images of untreated, APPJ-treated and APPJ/DBD-treated (DBD treatment time: 15 s) PLA, PLA/PAni, and PLA/PAni:CSA scaffolds; Figure S2: Optical emission spectrum of the argon plasma jet (APPJ) in CHL:DMF (8:2 v/v); Figure S3: Example of an N1s curve fitting of APPJ/DBD-treated PLA; Figure S4: Fluorescence microscopy images of PC-12 cells cultured on DBD-treated nanofibers for 10 days; Figure S5: Density of extended neurites from PC-12 cells cultured on PLA, PLA/PAni and PLA/PAni:CSA scaffolds; Table S1: Relative concentrations (%) of nitrogen-containing functional groups of APPJ/DBD-treated PLA nanofibers.
