# Supplementary material for: Polylactic Acid/Polyaniline Nanofibers Subjected to Pre- and Post-Electrospinning Plasma Treatments for Refined Scaffold-Based Nerve Tissue Engineering Applications
Source: Polymers (Basel). 2022 Dec 24;15(1):72. doi: 10.3390/polym15010072 (PMC9824446; doi:10.3390/polym15010072)
Supplement: Supplementary file 1 [file polymers-15-00072-s001.zip › polymers-2068063-SI.pdf]

Supplementary Material for

Polylactic acid/polyaniline nanofibers subjected to pre- and post-electrospinning plasma treatments for refined scaffold-based nerve tissue engineering applications

Yongjian Guo*, Rouba Ghobeira, Sheida Aliakbarshirazi, Rino Morent, and Nathalie De Geyter

Department of Applied Physics, Research Unit Plasma Technology (RUPT), Faculty of Engineering & Architecture, Ghent University, Sint-Pietersnieuwstraat 41, B4, 9000 Ghent, Belgium

*Corresponding author: yongjian.guo@ugent.be

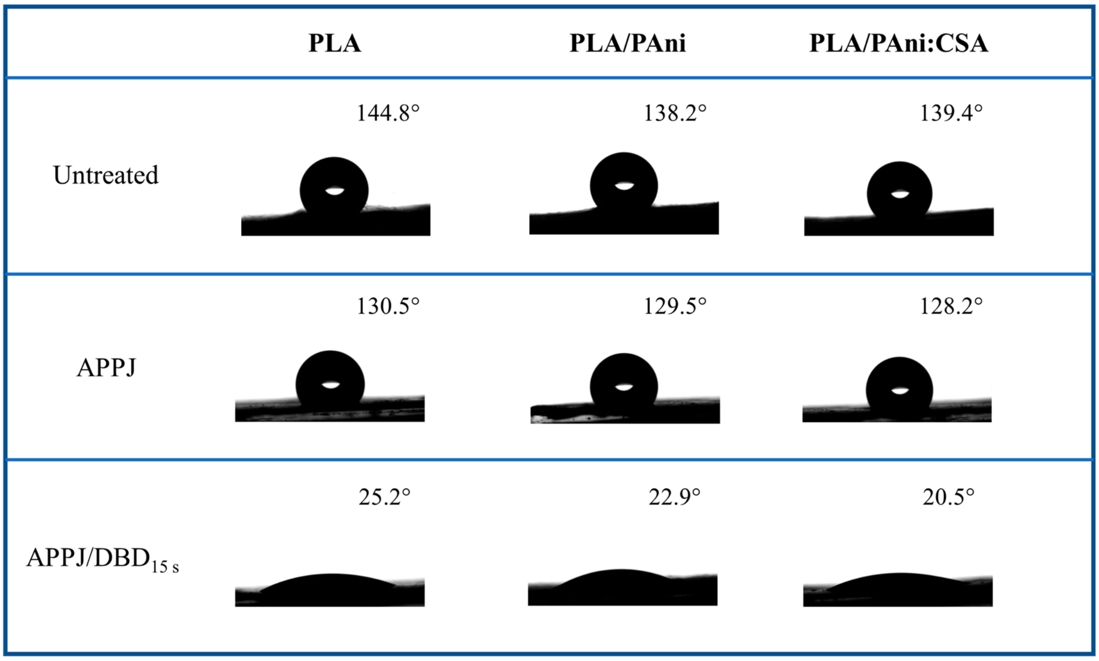

Figure S1 Representative WCA images of untreated, APPJ-treated and APPJ/DBD-treated (DBD treatment time: 15 s) PLA, PLA/PAni, and PLA/PAni:CSA scaffolds.

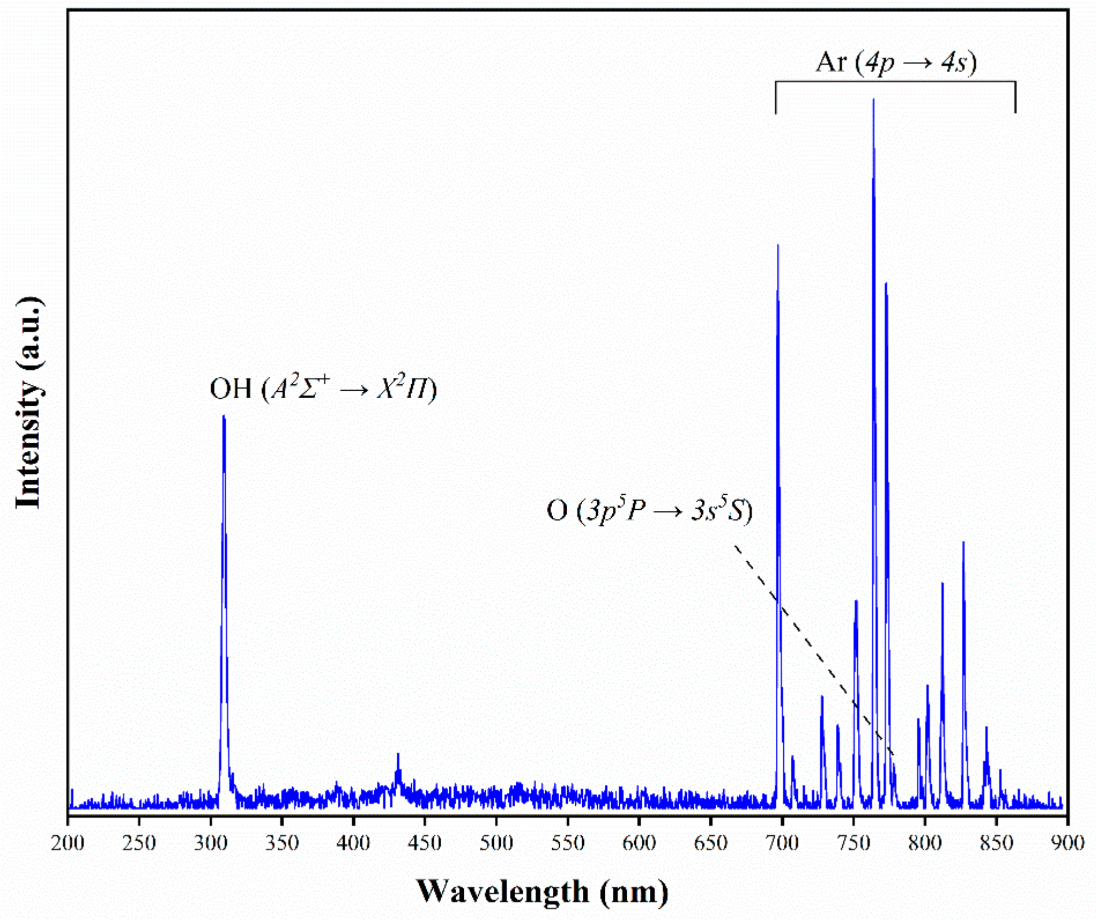

Figure S2 Optical emission spectrum of the argon plasma jet (APPJ) in CHL:DMF (8:2 v/v).

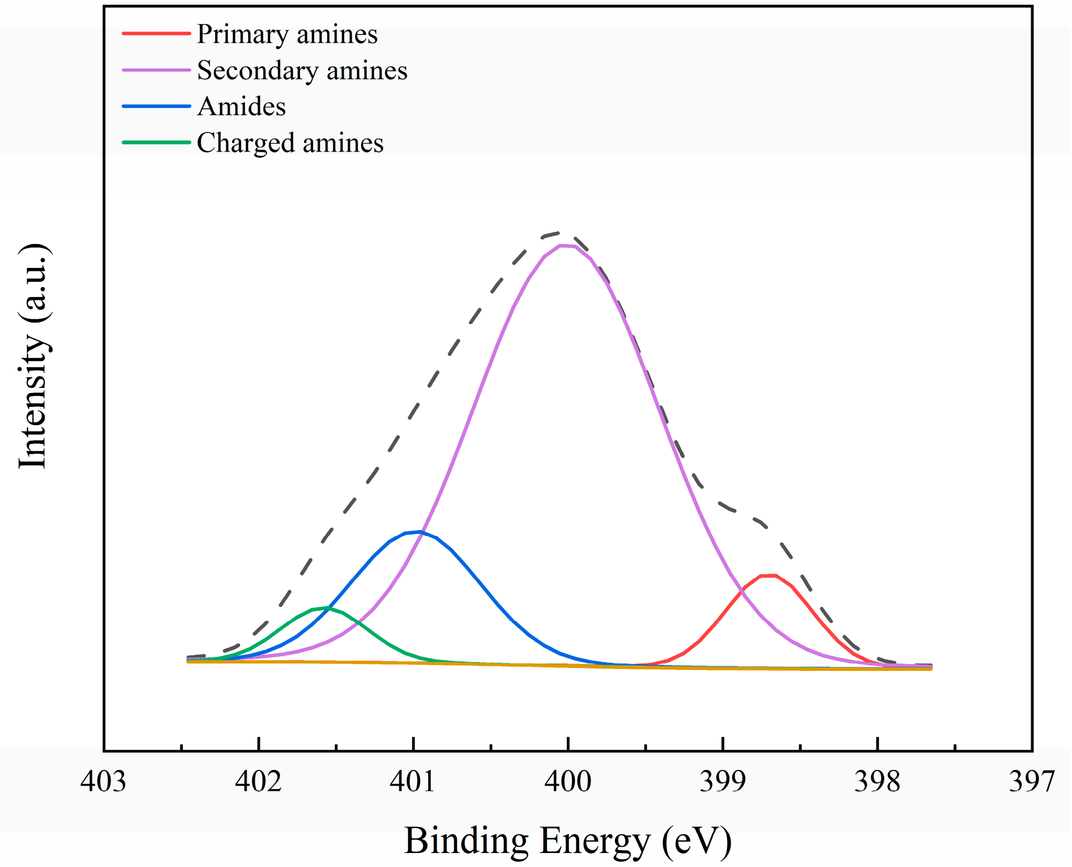

Figure S3 Example of an N1*s* curve fitting of APPJ/DBD-treated PLA.

Table S1

Relative concentrations (%) of nitrogen-containing functional groups of APPJ/DBD-treated PLA nanofibers.

Peak	primary amines (398.7 eV)	secondary amines (400.0 eV)	amides (400.9 eV)	charged amines (401.6 eV)
Concentration	7.4 ± 6.0	68.3 ± 10.4	16.2 ± 9.7	8.0 ± 6.2

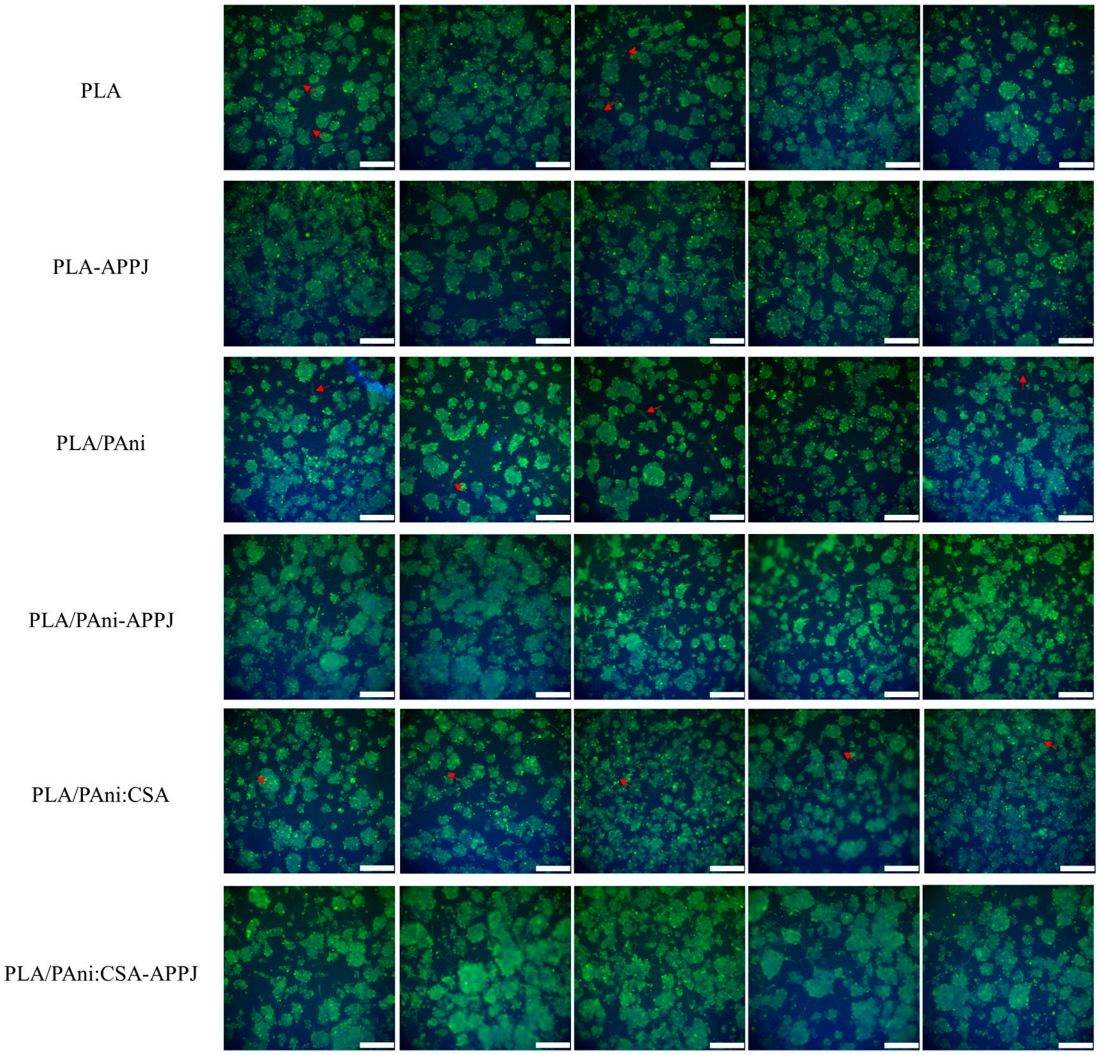

Figure S4 Fluorescence microscopy images of PC−12 cells cultured on DBD-treated nanofibers for 10 days (Scale bar: 500 μm).

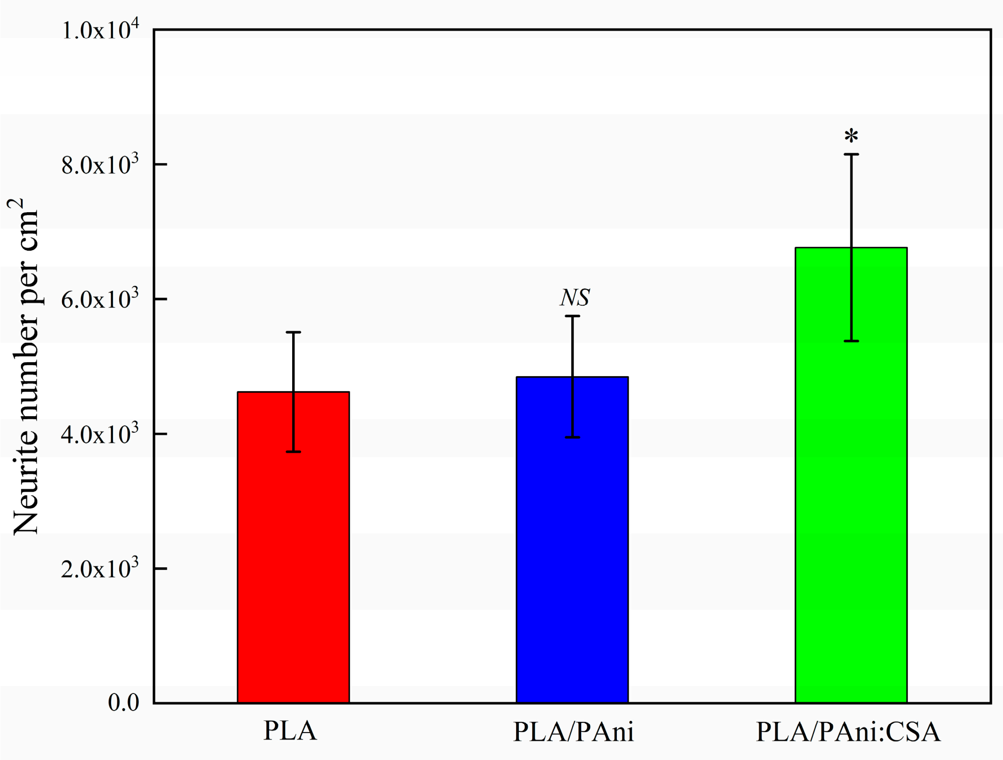

Figure S5 Density of extended neurites from PC−12 cells cultured on PLA, PLA/PAni and PLA/PAni:CSA scaffolds, $n \geq 5$, $*p < 0.05$ compared to PLA scaffolds, *NS*: not significant compared to PLA scaffolds.
